# Longitudinal Proteomic Signatures of Dupilumab and Tralokinumab Treated Atopic Dermatitis Patients

**DOI:** 10.1002/clt2.70203

**Published:** 2026-09-24

**Authors:** Wojciech Francuzik, Kristijan Pažur, Margitta Worm

**Affiliations:** ^1^ Division of Allergy and Immunology Department of Dermatology, Venereology and Allergology Charité—Universitätsmedizin Berlin Corporate Member of Freie Universität Berlin and Humboldt‐Universität zu Berlin Berlin Germany

**Keywords:** atopic dermatitis, biologic therapies, dupilumab, proteomics, tralokinumab

## Abstract

**Background:**

Atopic dermatitis (AD) is a chronic inflammatory disease driven by Type 2 (Th2) inflammation. Dupilumab (anti‐IL‐4Rα) and tralokinumab (anti‐IL‐13) are effective biologic therapies, but a direct comparison of their longitudinal effects on the systemic proteome is lacking. This exploratory study aimed to identify and to differentially analyze the proteomic signatures in AD patients treated with dupilumab versus tralokinumab.

**Methods:**

In this prospective, observational study, serum samples were collected from patients with moderate‐to‐severe AD at baseline and after 12 months of treatment with either dupilumab (*n* = 10) or tralokinumab (*n* = 10). A cohort of healthy controls (*n* = 12) was included for baseline comparison. Proteomic profiling was performed using a targeted 440‐protein array, and data were analyzed for differential expression and pathway enrichment.

**Results:**

At baseline, AD patients exhibited a distinct proteomic signature compared to healthy controls, characterized by a strong Th2 inflammatory profile and enrichment of the IL‐4/IL‐13 signaling pathway. After 12 months, both therapies reduced the core Th2 inflammatory signature with downregulation of key markers like CCL17. Dupilumab induced a broader immunomodulation, showing a more pronounced upregulation of Th1 associated proteins compared to tralokinumab. Furthermore, the therapies had opposing effects on tissue repair and metabolic proteins; dupilumab treatment led to a decrease in TNFSF11 and Sclerostin (SOST), while these were increased after tralokinumab treatment.

**Conclusion:**

Dupilumab and tralokinumab effectively target the Th2 axis in AD but their longitudinal systemic immune modulation differs in respect to Th1‐Th17 and tissue repair markers.

## Graphical abstract

Although both dupilumab and tralokinumab effectively suppress core Th2 inflammation in atopic dermatitis, they exhibit divergent, treatment specific protein modulation.

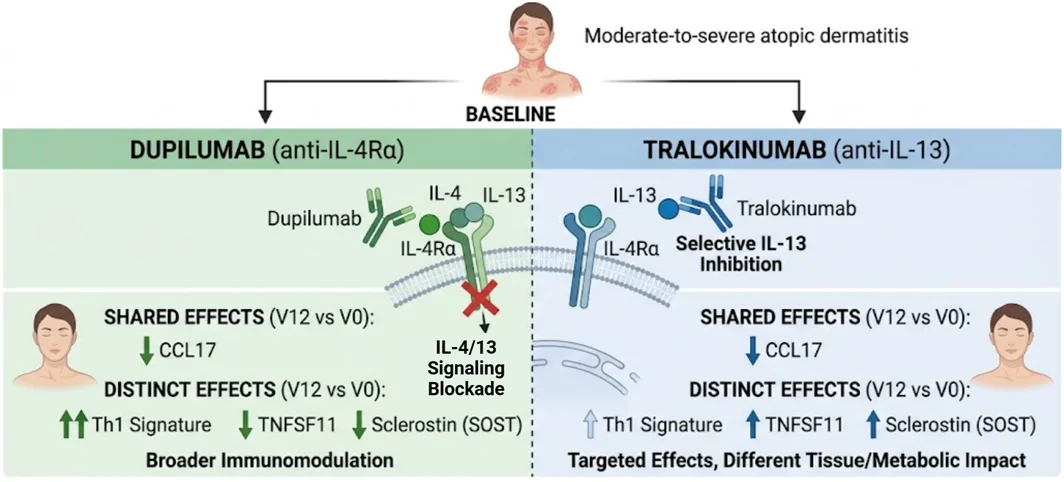

## 1 Introduction

Atopic dermatitis (AD) is a chronic inflammatory skin disease characterized by intense pruritus, eczematous lesions, and a relapsing‐recurring course. It affects a significant portion of the population, with estimates ranging from 10% to 13% in children and 6%–7% in adults [1]. AD significantly impacts quality of life, causing sleep disturbance, social isolation, and increased risk of secondary infections [2]. The pathogenesis of AD is complex and multifactorial, involving an interplay of genetic susceptibility, impaired skin barrier function, and immune dysregulation [1]. Genetic factors, such as mutations in the filaggrin gene (FLG), contribute to epidermal barrier defects leading to increased transepidermal water loss (TEWL) and allowing allergens and irritants to penetrate the skin and trigger immune activation [3]. This immune dysregulation is characterized by a predominantly type 2 helper T cell (Th2) response, along with contributions from other innate and adaptive immune cells, with increased production of cytokines like interleukin (IL)‐4, IL‐5, and IL‐13 [4]. These cytokines drive inflammation, promote IgE production, and contribute to the characteristic features of AD, such as pruritus and eczematous lesions.

Dupilumab, a monoclonal antibody targeting the shared receptor subunit of IL‐4 and IL‐13 (IL‐4Rα), has emerged as a highly effective treatment for moderate‐to‐severe AD [5]. By inhibiting IL‐4 and diminishing IL‐13 signaling, dupilumab reduces Th2 inflammation, improves skin barrier function, and is clinically highly effective. Tralokinumab, another biologic, specifically targets IL‐13, preventing its interaction with IL‐13Rα1 [6]. It is also clinically highly effective, though indirect comparisons in clinical studies resulted in less clinical efficacy in comparison to dupilumab after 16 weeks of treatment.

The variable clinical responses to biologics underscore the underlying complexity of AD. Increasing evidence suggests that AD, despite its common clinical presentation, represents a heterogeneous disease characterized by distinct pathophysiological mechanisms [7]. This heterogeneity likely contributes to the variability in treatment response observed with biologics. While some patients achieve complete or near‐complete clearance of their skin lesions, others experience only partial improvement. Identifying specific endotypes based on underlying molecular profiles, such as cytokine signatures, could hold the key predicting treatment response and facilitating personalized therapeutic strategies. Therefore, a longitudinal proteomic analysis comparing the effects of dupilumab and tralokinumab could provide crucial insights into their distinct mechanisms of action and identify potential biomarkers of treatment response.

### 1.1 Aim of the Study

This study aimed to analyze the longitudinal proteomic serum profiles from AD patients treated with either dupilumab or tralokinumab. We hypothesized that, despite both targeting the Th2 pathway, these biologics may promote distinct proteomic signatures reflecting their different molecular targets.

## 2 Materials and Methods

### 2.1 Study Design and Patient Cohort

We included 20 adult patients with moderate‐to‐severe atopic dermatitis (AD). Patients were eligible if they were 18 years or older on the day of inclusion, had a clinical diagnosis of AD according to the criteria of Hanifin and Rajka, an Investigator's Global Assessment (IGA) score of 3 or greater, a body surface area (BSA) involvement of 10% or greater, and had inadequate response to topical therapies, including topical corticosteroids and/or topical calcineurin inhibitors. Patients who had received prior systemic biologic therapy for AD, or other systemic immunosuppressants (e.g., janus kinase inhibitors, cyclosporine) within 4 weeks of baseline, were excluded.

Two treatment groups were prospectively included: (a) Dupilumab: 10 patients receiving dupilumab (Dupixent, Sanofi and Regeneron Pharmaceuticals; initial dose of 600 mg followed by 300 mg every other week); (b) Tralokinumab: 10 patients receiving tralokinumab (Adtralza, LEO Pharma; initial dose of 600 mg followed by 300 mg every other week). Patient allocation to treatment groups followed a sequential chronological recruitment strategy based on regional drug availability and regulatory approvals. Both cohorts were prospectively recruited using identical inclusion and diagnostic criteria. The dupilumab cohort was enrolled first following its clinical introduction. Upon completion of sample collection for this group, tralokinumab became available at our center; subsequent eligible patients initiating biologic therapy were sequentially allocated to the tralokinumab cohort under the same standard selection protocol. 12 healthy sex‐ and age‐matched individuals served as controls. Healthy controls were recruited and matched to the AD patients based on age (± 5 years) and sex. Patients were allowed to use concomitant topical corticosteroids and topical calcineurin inhibitors, provided the doses were stable for at least 4 weeks prior to baseline and remained stable throughout the study.

Clinical parameters were comprehensively assessed during ambulatory visits using objective disease severity measures SCORing Atopic Dermatitis (SCORAD), Investigators Global Assessment (IGA), Body Surface Area (BSA) and patient‐reported outcomes, which included Visual Analog Scales for itch and sleep loss (range 0–10), the Dermatology Life Quality Index (DLQI), and the WHOQOL‐BREF questionnaire, which evaluated quality of life domains.

The study was approved by the Charité Institutional Review Board (IRB), and informed consent was obtained from all participants.

### 2.2 Sample Collection and Processing

Serum samples were collected at baseline (V0) and 12 months (V12) for both treatment groups. Blood collection was performed between 2 and 4 p.m. using venipuncture and serum collection tubes (Vaccutainer, Becton, Dickinson & Co.). Within 1 hour of collection, blood was centrifuged at 1600 g for 5 minutes at 4°C in a swing‐out rotor centrifuge. Serum was then aliquoted and stored at −80°C until analysis. Samples were not thawed prior to being shipped frozen on dry ice to RayBiotech (Norcross, GA, USA) for analysis.

### 2.3 Proteomic Analysis

Proteomic profiling was performed by RayBiotech (Peachtree Corners, GA, USA) using the Quantibody Human Cytokine Array Q440 (Catalog #: QAH‐CAA‐440‐1), which quantifies 440 pre‐selected cytokines, chemokines, growth factors, and other proteins (Table S1). Briefly, serum samples (quadruplicates) were diluted and incubated with the array slides. After washing, biotinylated detection antibodies were added, followed by streptavidin‐conjugated Cy3 equivalent dye. Fluorescence signals were detected using a laser scanner. The array protocol followed the manufacturer's instructions, including sample preparation, incubation, washing, and chemiluminescence detection. Measurement data were provided by RayBiotech in pg/ml (concentration was calculated on luminenscence raw data that was fitted to a standard curve using 8 known concentrations per analyte) and further analyzed using the R statistical package with Bioconductor packages.

### 2.4 Data Analysis

The proteoDA package was used for data normalization, quality control, and differential expression analysis. VSN normalization of the data was selected as it best minimized technical variation and improved data distribution (Supporting Information S1: Figure S2). Values below the detection limit, reported as 0 pg/mL by RayBiotech, were replaced with half the limit of detection value for the respective analyte.

To evaluate treatment‐induced longitudinal changes while accounting for inter‐individual variability, differential expression analysis was conducted on an individual patient basis rather than cross‐sectional group averages. Linear mixed‐effects models were implemented using the limma package, incorporating Patient ID as a random effect. This framework explicitly models each subject's baseline proteomic level, calculating within‐patient differences prior to comparing treatment‐induced trajectories between cohorts. Proteins with a *p*‐value < 0.05 and an absolute log2 fold change > 1.5 were considered differentially expressed. A log2 fold change threshold of 1.5 was chosen to identify proteins with substantial changes in expression, balancing sensitivity and specificity. To account for multiple testing, *p*‐values for the baseline comparisons between healthy controls and atopic dermatitis patients were adjusted using the Benjamini‐Hochberg false discovery rate (FDR) procedure. Given the exploratory nature of this study and the focus on identifying potential protein signatures, *p*‐values were not adjusted for multiple comparisons in the direct longitudinal comparison between dupilumab and tralokinumab cohorts. This approach prioritizes the discovery of potential signals, but the findings should be considered hypothesis‐generating and require validation in a larger study. Differentially expressed proteins, ranked by t‐statistic, were further analyzed using the clusterProfiler for enrichment analysis incorporating the Reactome and MSigDB Databases.

## 3 Results

### 3.1 Patient Characteristics

Baseline demographic and clinical characteristics of the study participants are summarized in Table 1. The dupilumab group (*n* = 10) and tralokinumab group (*n* = 10) were generally comparable in terms of age, sex, and baseline disease severity, as assessed by SCORAD, IGA, and BSA. No significant differences were observed in DLQI scores between the AD groups or in any of the quality of life domains according to WHOQOL‐BREF questionnaire.

**TABLE 1:** Baseline demographic and clinical characteristics of atopic dermatitis patients treated with dupilumab or tralokinumab, and healthy controls.

Group	Dupilumab (*n* = 10)	Tralokinumab (*n* = 10)	Healthy controls (*n* = 12)	*p*‐value
Age (years)	33.4 ± 5.7	43.9 ± 18.8	36.8 ± 10	0.178^a^
Female sex (%)	40%	60%	58.3%	0.738^b^
BSA (%)	30 ± 18.5	30 ± 29.8	NA	0.849^c^
SCORAD	61.4 ± 9.1	54.9 ± 18.2	NA	0.821^c^
DLQI	19.5 ± 12.2	11 ± 2	NA	0.19^c^
Physical QOL	53.6 ± 18.8	67.9 ± 19.6	NA	0.623^c^
Psychological QOL	54.2 ± 15.6	45.8 ± 37.5	NA	0.967^c^
Social QOL	66.7 ± 31.2	58.3 ± 20.8	NA	0.369^c^
Environmental QOL	78.1 ± 18.8	65.6 ± 21.9	NA	0.412^c^

Abbreviations: NA, Not applicable; QOL, Quality of Life domains according to WHOQOL‐BREF.

^a^ ANOVA Test.

^b^ Fisher's Exact Test.

^c^ Mann‐Witney *U* Test.

### 3.2 Clinical Efficacy

**FIGURE 1:**
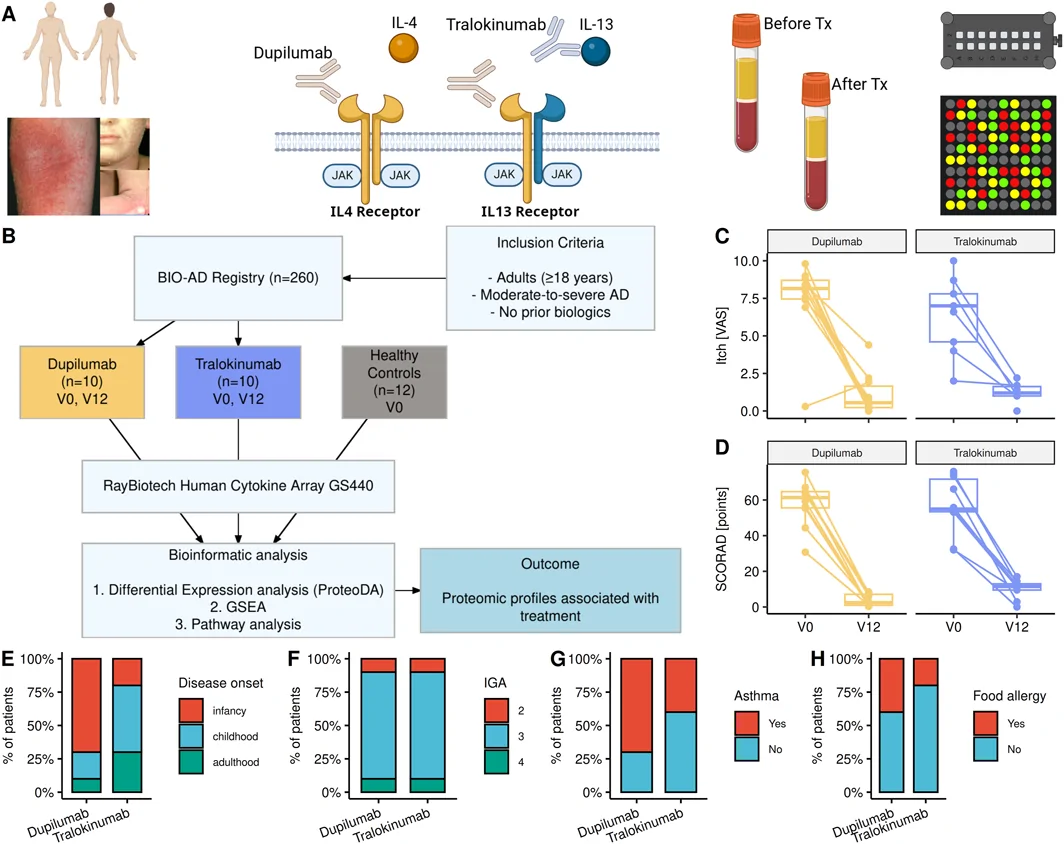
An overview of the study design, patient cohort, and clinical characteristics in atopic dermatitis patients treated with dupilumab or tralokinumab. (A) Concept of the study and a schematic of the drugs' mechanisms of action. (B) The study flow diagram of the included participants. (C–H) Clinical characteristics of the studied population.

Itch scores, as measured by a Visual Analog Scale (VAS), (dupilumab 8.15, IQR (7.5–8.8) and tralokinumab 7.00, IQR (5.4–8.6), Wilcoxon *p* = 0.27; Figure 1C) decreased after 12 months of therapy to 0.55, IQR (0.0–1.3) and 1.20, IQR (0.9–1.5) respectively. Similarly, baseline SCORAD scores were comparable between the two treatment groups on baseline (dupilumab = 61.35, IQR (56.8–65.9), tralokinumab = 54.90, IQR (45.8–64), Wilcoxon *p* = 0.85; Figure 1D) and decreased upon 12 months of therapy (2.45, IQR (0.0–5.5) and 11.50, IQR (9.8–13.2) respectively, Wilcoxon *p* = 0.85; Figure 1D).

The distribution of disease onset (infancy, childhood, adulthood) was not significantly different between the dupilumab and tralokinumab groups (χ^2^ *p* = 1; Figure 1E). The distribution of Investigator's Global Assessment (IGA) severity scores (0–3) was also not significantly different between the groups (χ^2^ *p* = 1; Figure 1F). The proportion of patients reporting asthma was not significantly different between the dupilumab and tralokinumab groups (χ^2^ *p* = 0.37; Figure 1G), nor was the proportion reporting food allergies (χ^2^ *p* = 0.63; Figure 1H).

### 3.3 Proteomic Profiles During Biologic Therapy

**FIGURE 2:**
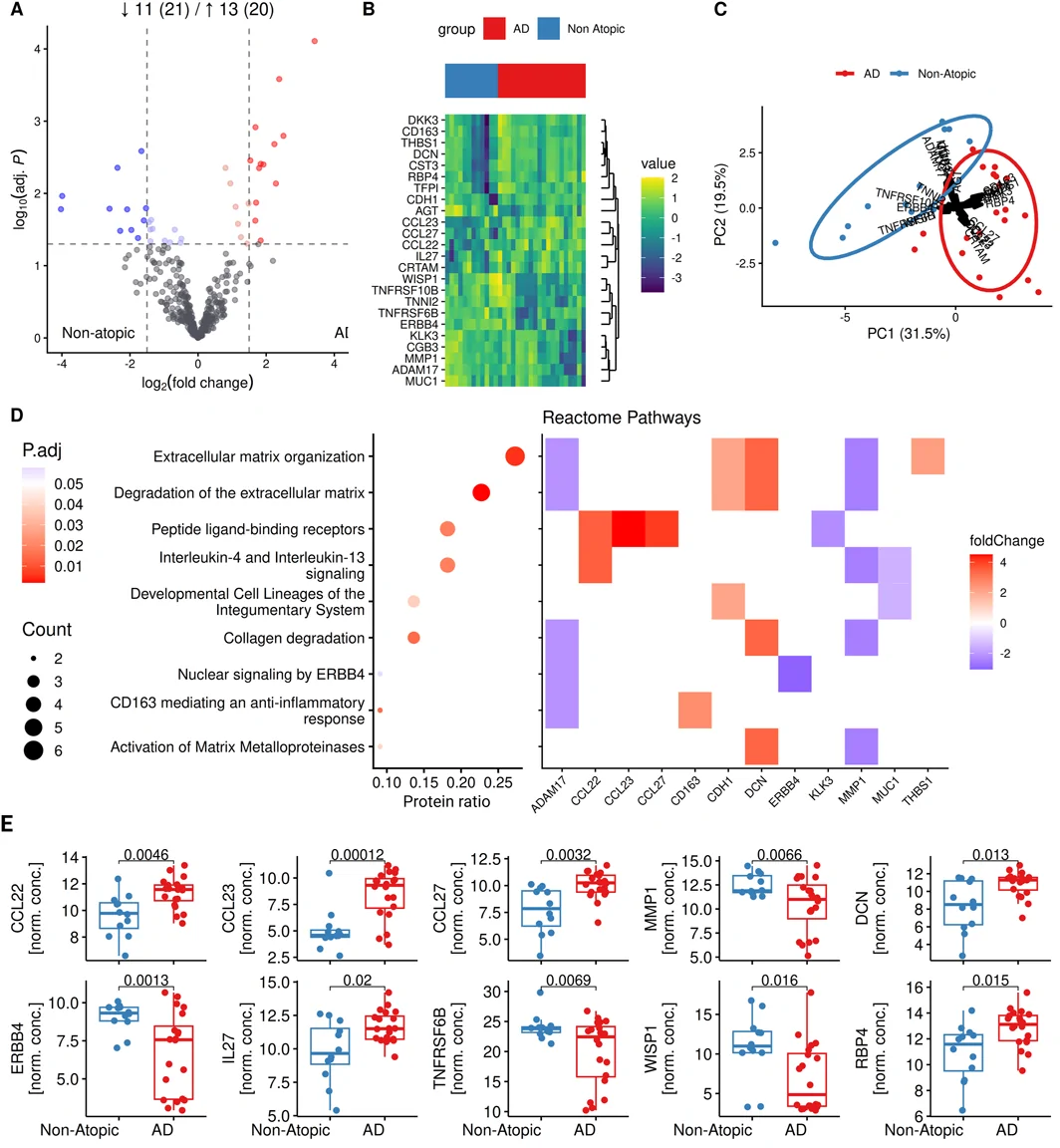
Proteomic analysis results in healthy individuals compared to atopic dermatitis patients. (A) Volcano plot of differentially expressed proteins, (log2FC > 1.5, *p* adj. < 0.05, shown in red). (B) Heatmap of differentially expressed proteins, (C) principial component analysis. (D) Pathway enrichment analysis (Protein ratio represents the proportion of significantly altered proteins identified within each enriched biological pathway), (E) individual box plot of protein normalized concentrations in two groups.

To investigate the systemic proteomic signature of atopic dermatitis and its potential reflection of the underlying Th2‐driven inflammation, we compared analyzed serum protein profiles between AD patients (*n* = 20) and healthy controls (*n* = 12) using a proteomic array broadly targeting potential mediators. The serum proteome from AD patients was consistent with a systemic inflammatory state. Proteins known to be associated with Th2 inflammation or its downstream signals were upregulated in AD patients, including CCL23, CCL27, CCL22. Conversely, downregulated proteins included Angiotensinogen (AGT), WNT1 Inducible Signaling Pathway Protein 1 (WISP1), and the receptor tyrosine kinase ERBB4 pathway (Figure 2A,B and E).

This distinct proteomic signature consistently segregated AD patients from healthy individuals, as visualized by the heatmap of differentially expressed proteins (Figure 2B) and principal component analysis (PCA; Figure 2C).

To connect these proteomic alterations to specific biological pathways implicated in AD pathogenesis, we performed pathway enrichment analysis (Figure 2D) using the Reactome database which indicated a significant involvement of 7 pathways. The analysis revealed enrichment of the “Interleukin‐4 and Interleukin‐13 signaling” pathway, a hallmark of the Th2‐mediated inflammation which directly links the observed systemic proteomic changes to the central pathogenic mechanism of AD. Furthermore, pathways related to “Extracellular matrix organization” and “Degradation of the extracellular matrix” were also enriched.

### 3.4 Comparative Analysis Between Dupilumab and Tralokinumab Responses

**FIGURE 3:**
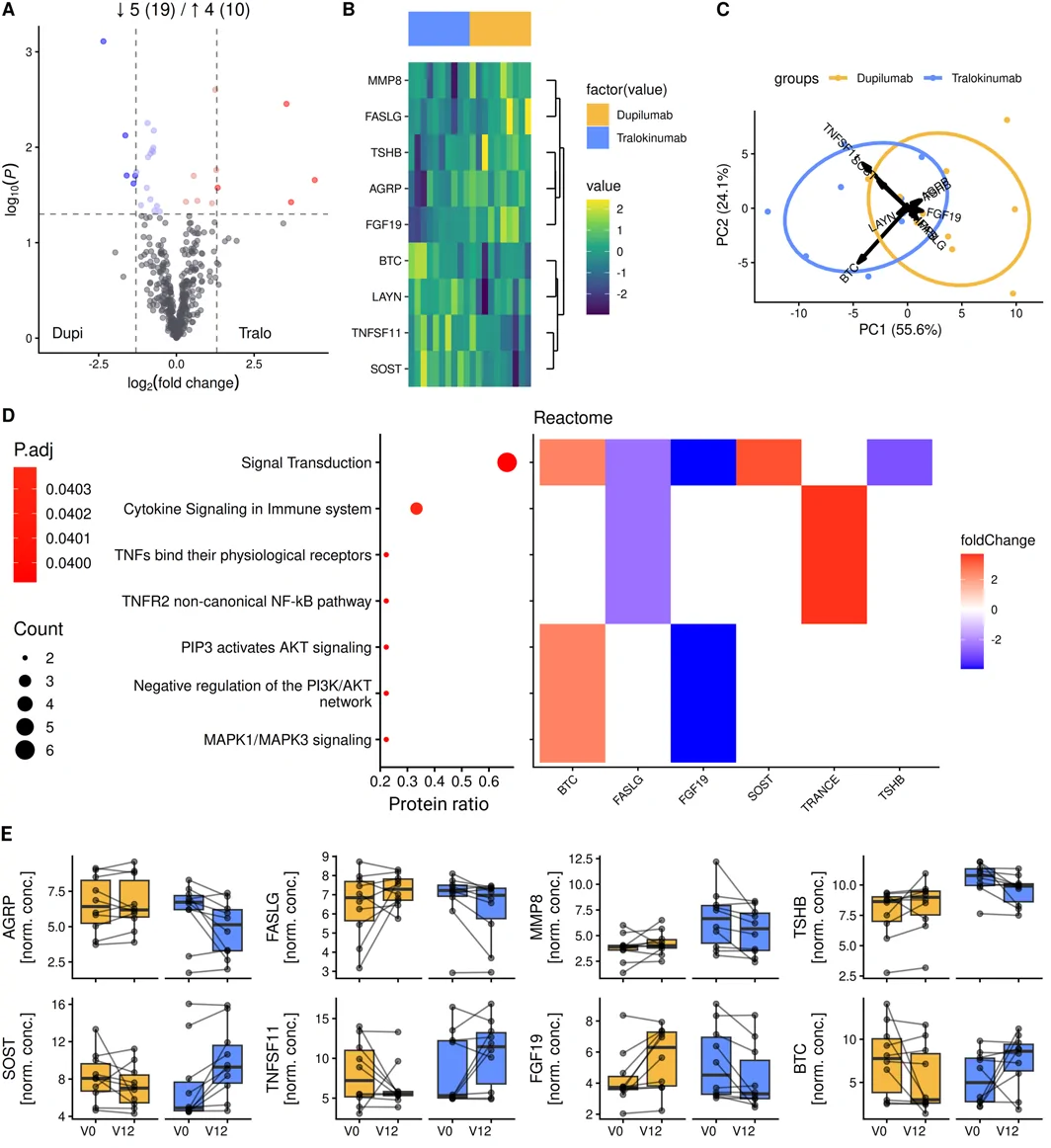
Proteomic analysis between Dupilumab and Tralokinumab Responses in atopic dermatitis patients. (A) Volcano plot of differentially expressed proteins, (log2FC > 1.5, *p* < 0.05, shown in red). (B) Heatmap of differentially expressed proteins, (C) principial component analysis. (D) Pathway enrichment analysis (Protein ratio represents the proportion of significantly altered proteins identified within each enriched biological pathway), (E) individual box plot of protein normalized concentrations in two treatment groups.

To evaluate the longitudinal proteomic effects of dupilumab and tralokinumab in AD patients, we assessed serum protein changes at 12 months (V12) with each treatment group taking V0 as baseline values (Figure 3). The volcano plot (Figure 3A) reveals distinct patterns of protein changes in regard to used biologic drug. 9 proteins were significantly altered (*p* < 0.05 and absolute fold change over 1.5) with 4 upregulated in the tralokinumab group and 5 upregulated in dupilumab group.

Figure 3B and 3E visualize these treatment‐dependent changes, displaying the relative abundance of significantly altered proteins across individual patients. While both treatments show evidence of protein modulation, dupilumab treatment was associated with a more pronounced upregulation of a cluster of proteins including MMP8, FASLG, FGF19, while tralokinumab treatment showed a more prominent upregulation of SOST and BTC. This visual distinction hints at differential mechanisms of action despite both targeting the Th2 pathway.

Principal Component Analysis (PCA; Figure 3C) further highlights the longitudinal proteomic shifts. Within both treatment groups, there is a separation between dupilumab and tralokinumab samples, although these differences were less pronounced than the ones seen for AD versus healthy individuals. Loadings analysis identifies SOST, BTC, and TNFSF1 as key contributors to the separation, suggesting their potential involvement in the treatment response.

To gain insights into the biological processes differentially affected by each treatment, we performed pathway enrichment analysis on the significantly altered proteins (Figure 3D). The dot plot (Figure 3D, left) reveals the differences in enrichment analysis between groups attributed to pathways related to ‘Signal Transduction’ and ‘Cytokine Signaling’, suggesting differences in the activation of TNF or PI3K/AKT signaling. The heatmap (Figure 3D, right) provides a granular view of the protein changes within these enriched pathways.

Tralokinumab treatment was associated with upregulation of TNFSF11 (RANKL, TRANCE), which function is to bind to RANK on cells of the myeloid lineage and function as a key factor for osteoclast differentiation and activation. In contrast, dupiumab treatment was associated with an upregulation of FGF19. These differing pathway enrichment and protein changes suggest that, while both biologics target Th2 inflammation, they may exert distinct downstream effects on other biological processes relevant to AD.

**FIGURE 4:**
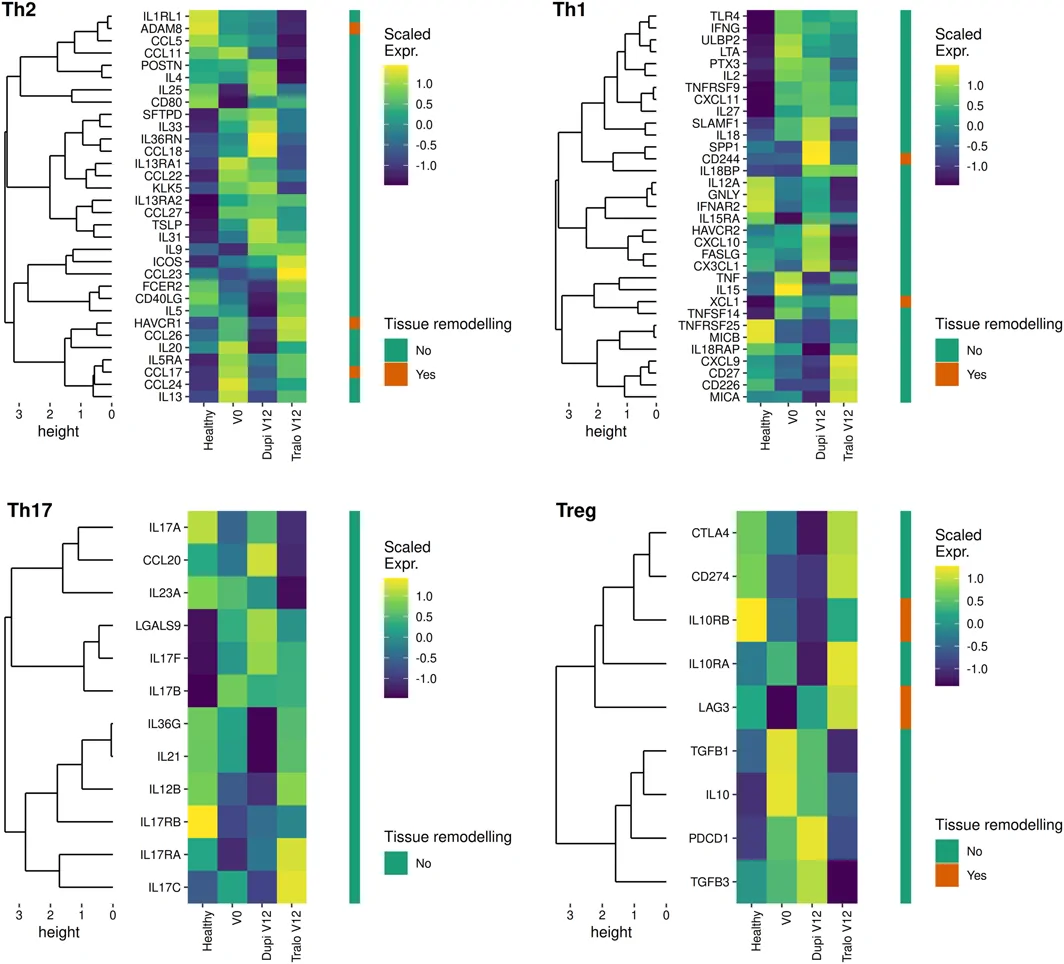
Heatmaps of protein expression in studied groups in regard to Lymphocyte T origin (targets predicted based on available gene sets and manual labeling).

We determined that although the CCL17 as well as IL13 decreased in both treated groups as expected, the decrease was more pronounced in patients receiving dupilumab (Figure 4). Interestingly, CD244 and IL1RL2 increased upon dupilumab treatment, while they remained unchanged in the tralokinumab group (Supporting Information S1: Figure S1).

On the other hand, tralokinumab treatment resulted in a more significant decrease of FGF19, E‐selectin (SELE) as well as ERBB2. TNFRSF11B, TNFRSF14 as well as IL18BP increased after treating with tralokinumab, but remained unchanged in patients receiving dupilumab (Supporting Information S1: Figure S1).

### 3.5 Cell Type Association

As T cell subsets are known to be disbalanced in AD we next determined the following T cell subtypes forming four distinct gene sets: (1) Th1, (2) Th2, (3) Th17 and (4) Tregs. Figure 4 illustrates the differences in mean expression of proteins in the samples. We observed differences in the Th2 set after 12 months of systemic therapy with either dupilumab or tralokinumab potentially showing a different pattern of activation of multiple protein groups (Figure 4).

A higher number of Th1 related proteins were upregulated after 12 months of dupilumab treatment than in patients treated with tralokinumab. The Treg associated proteins showed a similar pattern of expression in healthy and tralokinumab treated patients after 12 months of therapy, but not for the dupilumab treated group.

## 4 Discussion

This analysis represents one of the first published head‐to‐head comparisons of dupilumab and tralokinumab regarding their mechanism of action. Our findings confirm that both biological agents successfully target the Th2 axis, leading to the significant downregulation of key Th2‐associated proteins what is consistent with the known mechanisms of action for these monoclonal antibodies.

The observed suppression of Th2 activity aligns with prior research demonstrating that dupilumab treatment significantly and progressively reduces and normalizes the upregulated expression of Th2 signatures such as Chemokine (C‐C motif ligand) (CCL)17, CCL18, CCL22, and CCL26 in the lesional skin of AD patients [8]. Furthermore, dupilumab has been shown to suppress other Th2 mediators, including total IgE [9], periostin, and eotaxin‐3 [10]. In fact, serum CCL17 (TARC) is widely considered the most reliable biomarker for assessing clinical severity and monitoring the efficacy of AD treatment.

### 4.1 Receptor Types in IL4 and IL13 Signaling

The IL‐4 and IL‐13 cytokines signal through two distinct receptor complexes, each composed of different subunits. IL‐4 signals through the IL‐4 receptor (IL‐4R), which is a heterodimer composed of the IL‐4Rα chain and the common gamma chain (γc) [11]. IL‐13 signals through the IL‐13 receptor (IL‐13R), which is a heterodimer composed of the IL‐13Rα1 chain and the common IL‐4Rα chain [12]. The IL‐4Rα chain is shared between both receptors, allowing for cross‐signaling between IL‐4 and IL‐13 pathways. Their receptors are linked to different janus kinases. While the inhibition of IL‐4Rα by dupilumab blocks both IL‐4 and IL‐13 signaling (by targeting both: type 1 as well as type 2 receptors), tralokinumab specifically inhibits IL‐13 signaling by binding to the IL‐13Rα1 chain, preventing IL‐13 from interacting with the type 2 receptor [13]. Type 1 receptor is linked to JAK1 and JAK3, while Type 2 receptor is linked to JAK1, JAK2 and TYK2 [14].

We hypothesize that the partial blockade of Th2‐Axis by targeted IL‐13 signaling with tralokinumab allows for residual effects attributable to untargeted IL‐4 responses. Therefore, the visible changes in certain cytokines could be a result of IL‐4Rα signaling, which is associated with STAT6 but not with STAT3 (as this cascade is linked to the Type 2 cytokine receptor).

### 4.2 Differential Regulation of SOST, FGF19 and TNFSF11

In our exploratory analysis, candidate proteins differentiating the systemic profile between dupilumab and tralokinumab included Sclerostin (SOST) and Fibroblast Growth Factor 19 (FGF19), both of which contributed to the proteomic separation observed in Principal Component Analysis. Given the unadjusted nature of this screening array, these individual protein shifts should be interpreted with caution as preliminary signal discoveries rather than confirmed mechanistic differences.

SOST serum levels were found to be significantly higher in the “difficult‐to‐treat” severe AD patients group compared to patients whose disease was controlled with topical corticosteroids [15]. While our preliminary data indicated divergent trends in SOST regulation between treatment groups, whether IL‐4/IL‐13 axis modulation directly influences connective tissue or bone homeostasis regulators downstream remains hypothetical. As SOST is implicated in bone metabolism and its high level correlates with refractory disease, the downregulation seen with dupilumab may reflect a more comprehensive reversal of chronic systemic dysregulation, or a broader effect on tissue homeostasis compared to the selective IL‐13 blockade provided by tralokinumab.

FGF19 (Fibroblast Growth Factor 19) is characterized as an endocrine growth factor, part of the FGF family, which requires α‐ or β‐klotho for receptor activation [16]. While the exact role of increased FGF19 in AD remission following dupilumab treatment is unclear, high expression of FGF19 has been reported in the lesional skin of psoriasis patients^17^. FGF19 has been implicated in regulating keratinocyte biology and inflammatory processes (through Wnt/GSK‐3β/β‐catenin signaling [17]), although its exact involvement in maintaining immune balance or slowing disease progression requires further study. The differential increase of FGF19 only in the dupilumab group may suggests that the broader blockade of the IL‐4/IL‐13 signaling, including the IL‐4/JAK3 axis targeted uniquely by dupilumab, could lead to a distinct compensatory or regulatory pathway activation involving FGF19, which is not activated by the selective IL‐13 blockade of tralokinumab.

TNFSF11 (RANKL) plays a role in bone metabolism by driving osteoclasts. RANKL production is inhibited by tofacitinib (JAK1 and JAK3 inhibitor) in concentration dependent manner, and therefore suppresses osteoclast‐mediated structural damage to arthritic joints [18]. We also observed the decrease in TNFSF11 in dupilumab but not tralokinumab treated patients, which could be attributed to the blockade of type I receptor (specifically associated with JAK3). In regard, joint and bone pain are known side effects of dupilumab therapy [19], and might be reversed when patients switch to tralokinumab therapy [20].

Overall, while these individual protein candidates provide intriguing leads regarding pathway‐specific responses, definitive confirmation of divergent downstream pathways engaged by dupilumab and tralokinumab require targeted validation and FDR‐adjusted replication in independent, larger cohorts.

### 4.3 Immunomodulatory Signatures of Dupilumab and Tralokinumab

Although both biologics effectively suppress the Th2 axis, our proteomic analysis revealed slightly different immunomodulatory signatures associated with each treatment, suggesting differential downstream effects beyond Th2 inhibition. Dupilumab therapy resulted in the significant upregulation of Th1‐associated proteins. Specifically, a higher number of Th1 genes were upregulated after 12 months of dupilumab treatment compared to tralokinumab, which may be a consequence of dupilumab's more pronounced Th2 suppressive effect that we also observed on a cellular level [21]. Blockade of the IL‐4/IL‐13 pathway by dupilumab is suggested to lead to a polarization of the T cell repertoire toward pro‐inflammatory Th1 and Th17 pathways [22].

Proteins associated with regulatory T cells (Treg) exhibited a similar expression pattern in healthy individuals and in tralokinumab‐treated patients after 12 months of therapy. This observation, however, could not be made for the dupilumab‐treated group.

Dupilumab treatment was associated with the downregulation of tissue repair markers. This finding may be linked to the role of IL‐13, which is known to promote fibroblast‐mediated fibrosis and regulate down Matrix metalloproteinase‐13 (MMP‐13), a collagen degrader, thus contributing to the lichenification of skin in AD patients [23]. The difference in targets between dupilumab (blocking IL‐4/IL‐13) and tralokinumab (blocking only IL‐13) may therefore result in serum concentration of IL‐13 (lower levels after 12 months of dupilumab treatment, Figure 4 Th2 panel) and in effect influence outcomes related to tissue remodeling and repair.

### 4.4 Clinical and Personalized Medicine Implications

The observed divergence in proteomic profiles points toward distinct systemic effects of these two biologics, which could potentially influence patient management strategies. The concept of Th2 blockade potentially enhancing Th1/Th17 activity, leading to the manifestation of psoriasiform symptoms, has been previously reported [24]. Notably, cases of *de novo* psoriasis or psoriasiform eruptions have been reported with dupilumab [25]. The subtle mechanistic differences between the therapies may influence this risk; some patients who developed psoriasiform manifestations on dupilumab may not experience them when switched to tralokinumab [26].

### 4.5 Biomarker Limitations

Despite significant advances, AD remains a highly heterogeneous disease. The variable efficacy of targeted therapies, highlights the necessity of developing useful biomarkers to predict treatment success, especially given the considerable economic burden of these treatments. Several biomarkers are useful for monitoring disease activity (e.g., CCL17, CCL26/Eotaxin‐3, and LDH, which are suggested to be better than CCL17/TARC for assessing severity [27]). While no blood or serum biomarkers are currently known to reliably predict good or poor treatment outcomes in AD patients receiving biologics, we have recently identified that the baseline CCL17/E‐selectin ratio may hold predictive value for therapeutic response to dupilumab [28].

The proteomic signatures identified in this exploratory study, revealing the divergent effects of IL‐4/IL‐13 dual blockade versus selective IL‐13 blockade, are hypothesis‐generating and require validation in a larger study. Future work should focus on exploring the functional implications of the identified proteins and investigating their potential as predictive biomarkers for treatment response in AD.

Ultimately, achieving precision medicine necessitates the creation of comprehensive registries linking laboratory findings to objective clinical scores in order to guide therapeutic choice. As the Real‐World Clinical Studies (RWCS) are gaining popularity, there is a chance that we will be able to achieve this step in the future.

### 4.6 Limitations and Future Directions

Our study has inherent limitations, including the small sample size of the cohorts and the potential for confounding factors inherent in a real‐world, observational study design. The analysis focused on identifying potential protein signatures, and therefore, *p*‐values were not stringently adjusted for multiple comparisons, prioritizing discovery over strict statistical confirmation. Serum proteomics might not perfectly reflect the protein changes in lesional skin, although they are easier to obtain longitudinally. Furthermore, our cohort analyzed only adult patients of European descent, and due to differences in AD presentation based on age and ethnicity, the findings may not be broadly extrapolated without further investigation in diverse cohorts.

## 5 Conclusion

In conclusion, this study provides a comprehensive proteomic analysis of the systemic effects of dupilumab and tralokinumab in patients with atopic dermatitis. Our data confirm that both biologics effectively target the Th2 axis, leading to significant downregulation of key Th2‐associated proteins. However, dupilumab appears to induce a broader immunomodulatory effect, characterized by the significant upregulation of Th1‐associated proteins and the downregulation of specific tissue repair markers.

These divergent proteomic patterns suggest that while both therapies are effective in managing clinical AD, they exert distinct systemic effects—likely attributable to the differential blockade of IL‐4 versus selective IL‐13 inhibition. Given the exploratory nature of this study, these findings should be considered hypothesis‐generating. Future validation in larger cohorts is essential to determine if these systemic signatures can serve as predictive biomarkers to guide personalized treatment strategies and optimize patient outcomes.

## Author Contributions

**Wojciech Francuzik:** investigation, writing – original draft, methodology, validation, visualization, writing – review and editing, software, formal analysis, data curation. **Kristijan Pažur:** investigation, writing – original draft, methodology, validation, visualization, writing – review and editing, formal analysis, data curation. **Margitta Worm:** conceptualization, funding acquisition, writing – original draft, writing – review and editing, supervision, investigation, data curation.

## Funding

This study was funded by a research grant from LEO Pharma and Sanofi to MW and by the Deutsche Forschungsgemeinschaft (DFG, German Research Foundation) as part of the clinical research unit (CRU339): Food allergy and tolerance (FOOD@) – 409525714.

## Ethics Statement

The study was approved by the Charité Institutional Review Board (EA1/122/19).

## Consent

Patients and healthy individuals participating in this trial provided written informed consent prior to participation, in line with the Declaration of Helsinki.

## Conflicts of Interest

M. Worm has received scientific research grants from Sanofi and LEO Pharma. The remaining authors (W. Francuzik and K. Pažur) declare no potential conflicts of interest relevant to this article.

## Supporting information


Supporting Information S1

**Table S1:** Assay characteristics, detection limits, and quantification metrics for the targeted 440‐protein serum panel.

## Data Availability

The raw proteomic data and R code used for analysis will be deposited in the Figshare database (10.6084/m9.figshare.33144089) upon manuscript acceptance.
