# Supplementary material for: Longitudinal Proteomic Signatures of Dupilumab and Tralokinumab Treated Atopic Dermatitis Patients
Source: Clin Transl Allergy. 2026 Sep 24;16(9):e70203. doi: 10.1002/clt2.70203 (PMC13612374; doi:10.1002/clt2.70203)
Supplement: Supplementary file 1 [file CLT2-16-e70203-s002.docx]

# Supplement

Figure S1: Expression of 6 most differentially expressed proteins in regard to baseline visit (V0) in A) dupilumab- and B) tralokinumab-treated atopic dermatitis patients.

Figure S2: The Quality control and normalization procedure for choosing VSN as the normalization method.
